# Brazilian Portuguese version of the revised upper-limb module: cross-cultural adaptation and validation

**DOI:** 10.1055/s-0045-1805072

**Published:** 2025-03-19

**Authors:** Mariana Cunha Artilheiro, Juliana Rodrigues Iannicelli, Graziela Jorge Polido, Tatiana Ribeiro Fernandes, Leandro Augusto de Almeida, Rodrigo Holanda Mendonça, Clara Gontijo Camelo, Cristiane Araújo Martins Moreno, Edmar Zanoteli

**Affiliations:** 1 Universidade de São Paulo, Faculdade de Medicina, Departamento de Neurologia, São Paulo SP, Brazil.

**Keywords:** Muscular Atrophy, Spinal, Validation Study, Disability Evaluation, Upper Extremity

## Abstract

**Background** With the emergence of new therapies for spinal muscular atrophy linked to chromosome 5q (SMA-5q), capturing motor function changes in the upper limbs (ULs) become crucial for assessing treatment efficacy and monitoring changes in the disease. The translation and cultural adaptation of the revised upper-limb module (RULM) into Brazilian Portuguese will enable implementation in clinical settings and facilitate the conduction of national studies.

**Objective** To translate, cross-culturally adapt, and validate the RULM to Brazilian Portuguese.

**Methods** The study was conducted in two phases: translation and cross-cultural adaptation, and the reliability assessment. Both phases followed recommendations from international guidelines. The analysis of the psychometric properties was performed with 21 individuals with SMA-5q, all of whom were at least 30-months-old and capable of sitting independently. Statistical analyses were performed using Cohen's Kappa analysis (K) and the intraclass correlation coefficient (ICC).

**Results** The interrater agreement was considered excellent for items A to T (K > 0.81) and good (0.61–0.80) for items G and M. The reliability analysis showed an ICC of 0.998, indicating an extremely satisfactory level.

**Conclusion** The Brazilian Portuguese version of the RULM has been shown to be valid and reliable for the assessment of SMA-5q individuals older than 2 years of age who could sit.

## INTRODUCTION

Spinal muscular atrophy linked to chromosome 5q (SMA-5q) is an autosomal recessive progressive neurodegenerative disorder caused by mutations in the *SMN1* gene. 1 2 The disease leads to different grades of muscle weakness, hypotonia, and bulbar and respiratory insufficiency.

Patients are typically classified into at least three clinical forms based on their maximum motor ability and age of onset. 3 Type-I SMA-5q is the most common form and is characterized by an early onset (0–6 months of age), and the children cannot sit unaided. Type-II SMA-5q manifests between 6 and 18 months of age and affected children cannot walk unaided. Type-III SMA-5q typically starts after the second year of life, and affected individuals can walk unaided. 3 Effective disease-modifying therapies, such as *SMN1* gene replacement (onasemnogene abeparvovec) and those acting on the regulation of exon 7 splicing in *SMN2* (nusinersen, risdiplam), have already been approved by leading international regulatory agencies and the Brazilian Health Regulatory Agency (Agência Nacional de Vigilância Sanitária, ANVISA, in Portuguese).

Regardless of mobility level, the upper limb (UL) muscle strength is reduced and progressively weakens over time. 4 5 6 7 The most notable decline in UL function likely occurs during early adolescence in types II and III. 8 The reduction in muscle strength results in the use of compensatory mechanisms and also in difficulties in task performance, 9 10 11 leading to reduced independence, social participation, and quality of life. 12 13

In recent years, the assessment of UL function in children and adults with SMA has been extensively studied. With the emergence of new therapies, capturing alterations in UL function has become crucial to assess treatment efficacy and monitor changes caused by disease progression. 8 14

There was full consensus among expert clinicians that the revised upper-limb module (RULM) is the most robust scale for assessing UL function in SMA-5q. The tool effectively captures a wide spectrum of functional abilities across of ambulatory and nonambulatory patients and indicates an overall motor function. The assessment of relevant activities and the ability to score them accurately is important not only in clinical practice and rehabilitation but also in clinical trials. 15

The RULM has been used in numerous longitudinal natural history studies in combination with other disease-specific outcome measures, such as Hammersmith functional motor scale expanded (HFMSE), to monitor symptoms progression across all SMA-5q types. 8 The HMFSE 16 and the Children's Hospital of Philadelphia Infant Test of Neuromuscular Disorders (CHOP INTEND), 17 two of the most clinically important scales designed to evaluate gross motor ability in SMA-5q, and widely used in clinical trials and real-life studies, were recently translated and validated for the Brazilian population. However, neither of these scales is specific for UL assessment. The translation and cultural adaptation of the RULM into Brazilian Portuguese will enable its widespread implementation in clinical settings and facilitate the conduction of national studies, especially in the current treatment era, which is changing the natural history trajectories of patients with SMA-5q.

Thus, the aim of the present study was to translate, cross-culturally adapt, and validate the RULM so that it becomes a translated and validated scale for Brazilian Portuguese.

## METHODS

### Design

This observational study using a cross-sectional design was approved by the Ethics Committee of Hospital das Clínicas da Faculdade de Medicina da Universidade de São Paulo (HCFMUSP; under process number 3.520.910). Initially, all individuals or their legal guardians signed the permission or consent form before data collection. Subsequently, the study began, and participants were enrolled according to recommendations from international guidelines and previous studies. 18 19

**Figure 1:**
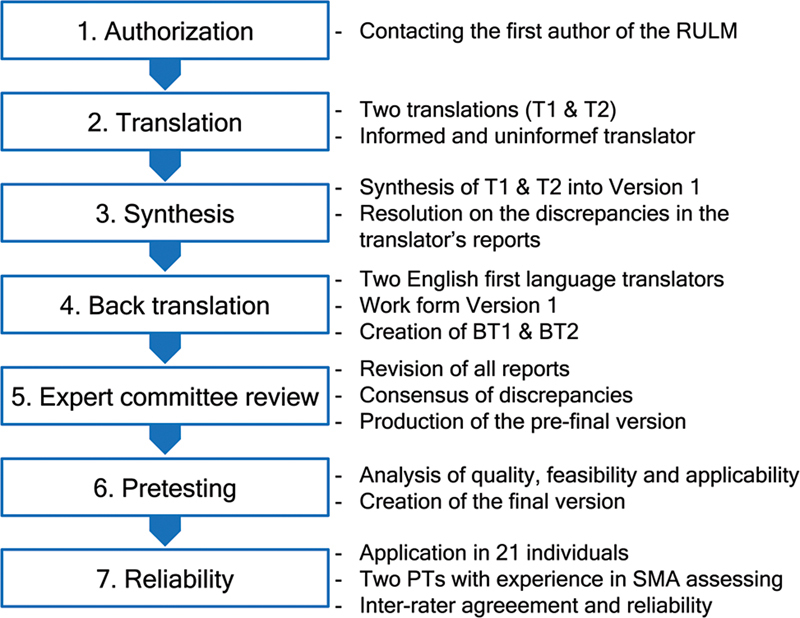
Stages of the translation and cross-cultural adaptation to Brazilian Portuguese of the revised upper-limb module.

The study was conducted in two phases: translation and cross-cultural adaptation of the RULM to Brazilian Portuguese and the reliability test. Both phases, following predetermined stages, are depicted in Figure 1 .

### Revised upper limb module (RULM) description

The RULM is an instrument revised and updated from the original upper-limb module (ULM), 4 which was developed by clinicians, physical therapists (PTs), researchers, and patient advocacy groups to capture the progressive muscle weakness in young children, as well as in both ambulant and nonambulant individuals with SMA-5q. The ULM was composed of nine items developed to assess the functional ability of the proximal muscles—complete the path, lift and transfer weight, bring the coin to a cup on a table, bring weight from lap to eye level and at arm length, take coin above shoulder height and bring on the table, push button, open ziploc, bring weight on shoulder height, and lift can to mouth.

The revised version, RULM, consists of 20 items, including an entry item (A) designed to identify the maximum motor ability. However, this item does not contribute to the total score. The 19 scorable items (B through T) assess different functional domains (such as shoulder, elbow, and wrist movements) and are rated on a 3-point system scale: 0 (unable), 1 (able with modification), and 2 (able without difficulty). The maximum possible score is 37 points. 15 The scale exhibits good reliability and validity 15 20 and is now extensively utilized in multicenter research and clinical trials. 8 21

### Translation and cross-cultural adaptation of the RULM to Brazilian Portuguese

The first stage involved contacting Elena S. Mazzone, the first author of the RULM development study, to obtain authorization to translate the instrument into Brazilian Portuguese.

The second stage involved two translations of the original RULM from English to Brazilian Portuguese. This process was performed by two independent professionals: an informed and an uninformed translator. Both translators were bilingual, with Portuguese as their native language and fluency in English. This resulted in two independent translations (T1 and T2).

The synthesis process of the T1 and T2 to produce version 1 (the first Brazilian Portuguese version) constituted the third stage. Both translators and the main investigator participated in discussions to resolve discrepancies between the reports.

To ensure that version 1 accurately reflected the exact content of the original RULM instrument, two back-translations were performed from this document (BT1 and BT2). This fourth stage was performed by two professionals whose native language is English and who were uninformed about the scale.

For the fifth stage, the Expert Committee Review revised and analyzed all the study reports (original version, T1, T2, Version 1, BT1, and BT2). To ensure that the discussion resulted in a prefinal version consistent with the original version, a face-to-face meeting was conducted with a committee of three PTs. These PTs had completed face-to-face RULM training, and each had at least 15 years of practice experience in assessing SMA individuals. The PTs were specifically recommended by the RULM's first author. The committee discussed the equivalences between the versions – whether semantic, idiomatic, cultural, or conceptual – and evaluated whether terms were suitable for all regions of the country. Terms or phrases considered inappropriate were replaced without altering the intended meaning. A consensus was reached on discrepancies, and changes to terms or expressions were discussed accordingly.

In the pretesting stage, the final stage, the prefinal version was administered to four individuals with SMA-5q, 16 who were treated at HCFMUSP. This was done by another PT to identify and correct any potential errors missed in previous stages, as well as to assess the quality, feasibility, and applicability of the RULM prefinal version. Out of the four individuals, three were male and one was female; three were diagnosed with type II and one with type III SMA-5q. All participants were nonambulatory. The mean age of the pretest participants was 17.75 ± 9.64 years, and the mean RULM score was 19.5 ± 9.3 points. After administering the pretest, the translated RULM version underwent a final review by the committee to ensure that any potential translation or grammatical errors were addressed. Once the final version was developed, it was returned to the RULM's first author.

### Reliability

The second phase tested interrater reliability and agreement.

To analyze these psychometric properties, 21 individuals with SMA-5q out of the pretesting were selected based on convenience and meeting the following inclusion criteria: a genetic test confirming the diagnosis of SMA-5q, an age of at least 30 months, and the ability to sit independently.

A single PT filmed and assessed each individual using the translated RULM Version. Two others, with extensive experience in neuromuscular disorders and SMA assessment and trained with the original instrument, independently analyzed the videos. Scores for each item and the final score were recorded. This approach was based on a recent study that performed the translation and cross-cultural adaptation of another important instrument for assessing SMA patients, the CHOP INTEND scale. 17 The objective was to avoid multiple assessments in a short period and to prevent muscle fatigue.

### Statistical analysis

Descriptive data analysis was performed to characterize the sample. Tests were run on the IBM SPSS Statistics for Windows (IBM Corp., Armonk, NY, USA) software, version 26.0.

Cohen's Kappa analysis was applied to investigate the agreement between two evaluators in assessing the RULM items. Interrater agreement for each item was classified as weak (0.0–0.2), slight (0.21–0.40), moderate (0.41–0.60), good (0.61–0.80), and excellent (0.81–1.0). 22

Interrater reliability analysis was calculated using the intraclass correlation coefficient (ICC) and its 95% confidence intervals (95%CIs), based on the total score. Reliability was classified as low if the ICC was below 0.40, moderate between 0.4 and 0.75, and high if the ICC was above 0.75. 23 24

Results were considered significant if the *p* -value was below 0.05. Thus, a significance level of 5% was adopted.

## RESULTS

A few inconsistencies were found after the translations and back-translations. Some words and terms were simply replaced to make the text more comprehensible without altering the original meaning. Regarding divergence between the translators, the researchers discussed and selected the most appropriate term. The consensus for each term and its respective equivalence is presented in Table 1 . For example, weight sand and OTLS were identified as cultural equivalence. Instead of OTLS, a well-known term in Brazil, the translation “órtese torácica, lombar e sacral” is less usable and practical. We decided to translate it as “colete” or “órtese torácica” to cover any type of trunk orthosis the individual may use. The same criteria were applied to weight sand. While weight sand is easily understood, “caneleira de peso” is the more commonly used term.

**Table 1:** Stages of the translation and cross-cultural adaptation to Brazilian Portuguese of the revised upper-limb module

Original term/word	Synthesis of T1 and T2	Specialist number one	Specialist number two	Defined term/word	Equivalence
Continuum of ability	Continuum of capability	Sequência de habilidades	Sequência integrada	Sequência de habilidades	Semantic
Equipament required	Equipamentos exigidos	Equipamentos necessários	Equipamentos exigidos	Equipamentos necessários	Semantic
Adjustable table top	Tampo de mesa ajustável	Mesa ajustável	Mesa com tampo regulável	Mesa com regulagem de altura	Semantic
Plastic cups (vending cup)	Copos plásticos (copos de venda)	Copos de plástico fime	Copos plásticos descartáveis	Copos plásticos descartáveis e firmes	Cultural
Metal cooking weight	Peso de cozinha de metal	Peso padrão de balança	Peso de cozinha de metal	Peso padrão para balança	Cultural
Plain A4 paper	Papel A4 comum	Folha sulfite comum	Papel sulfite comum	Papel sulfite (A4 comum)	Cultural
Sand weight	Peso de areia	Caneleira	Peso de areia	Peso de areia (caneleira)	Cultural
Loose clothing	Roupas largas	Roupas confortáveis	Roupas largar	Roupas confortáveis	Semantic
TLSO	Órtese torácica	Órtese torácica/colete	Órtese torácica/colete	Órtese torácica/colete	Cultural
Less able individuals	Indivíduos menos capazes	Indivíduos mais fracos	Indivíduos menos capazes	Indivíduos mais fracos	Semantic
Most able individuals	Indivíduos mais capazes	Indivíduos mais fortes	Indivíduos mais capazes	Indivíduos mais fortes	Semantic
Elbow to shoulder height	Altura do cotovelo ao ombro	Cotovelos na altura dos ombros	Cotovelos na altura dos ombros	Cotovelos na altura dos ombros	Semantic
No useful function of hands	Sem função útil das mãos	Sem uso funcional das mãos	Sem função útil das mãos	Sem uso funcional das mãos	Semantic
Only by flexing the elbow	Apenas flexionando o cotovelo	Apenas com o cotovelo fletido	Apenas se usar a flexão do cotovelo	Apenas com o cotovelo flexionado	Semantic
Tokens on the table in front the individual	Fichas à frente do indivíduo na mesa	Fichas na mesa em frente ao indivíduo	Fichas à frente do indivíduo na mesa	Fichas na mesa em frente ao indivíduo	Semantic
Hold all tokens in their hand	Ter todas as fichas na mão	Manter todas as fichas na mão	Segurar todas as fichas na mão	Segurar todas as fichas na mão	Semantic
Cup placed	O copo está colocado	O copo está colocado	O copo deve ser posicionado	O copo deve ser posicionado	Semantic
Bent or extender	Dobrado ou estendido	Fletido ou estendido	Em flexão ou extensão	Em flexão ou extensão	Semantic
Pushing it and hard enough	Ao apertar o botão com força suficiente	Apertando o botão forte o suficiente	Apertando o botão com força suficiente	Apertando o botão forte o suficiente	Semantic
Folded in 2/in 4	Dobrado em 2/em 4	Dobrado em 2/em 4	Dobrado em 2/em 4 partes	Dobrado em 2/4 partes	Semantic
Ziploc container	Recipiente Ziploc	Recipiente Ziploc	Pote Ziploc	Pote Ziploc	Semantic
In one motion	Um movimento só	Em um único movimento	Em um movimento único	Em um movimento único	Semantic
Block	Bloquear	Não permitir	Não permitir	Não permitir	Semantic
Straight elbows	Cotovelos retos	Cotovelos retos	Cotovelos esticados	Cotovelos esticados	Semantic
Preferred no arm rests on chair	Sem apoio para os braços na cadeira é preferível	É preferível uma cadeira sem apoio para braços	Sem apoio para os braços na cadeira é preferencial	Preferencialmente sem o apoio de braços na cadeira	Semantic

Abbreviations: T1, translation 1; T2, translation 2; TLSO, thoracic-lumbar-sacral orthosis.

Sample characteristics are displayed in Table 2 . One individual was diagnosed with type-I (4.76%), 12 with type-II (57.14%), and 8 with type-III (38.10%) SMA-5q. There were 12 female individuals (57.14%), and 9 male (42.86%). The mean age of the patients was of 13.85 ± 13.64 years, and most were nonambulant (80.95%).

**Table 2:** Characteristics of the study sample

Patient ( *N* = 21)	SMA-5q type	Gender	Age (years)	Walker
1	2	Female	9	No
2	3	Male	9	Yes
3	3	Male	26	No
4	2	Female	4	No
5	3	Female	9	Yes
6	2	Male	10	No
7	2	Female	9	No
8	2	Female	4	No
9	2	Female	4	Yes
10	2	Female	4	No
11	2	Male	4	No
12	2	Female	7	No
13	2	Female	15	No
14	2	Female	14	No
15	1	Female	5	No
16	3	Male	9	No
17	3	Male	11	No
18	3	Male	56	No
19	3	Male	31	Yes
20	3	Female	41	No
21	2	Male	10	No

Abbreviation: SMA-5q, spinal muscular atrophy linked to chromosome 5q.

Table 3 shows the inter-rater agreement, which was considered excellent for items A to T (K > 0.81), except for item G (K = 0.80) and for item M (K = 0.77), which were rated as good (0.61–0.80).

**Table 3:** Inter-rater agreement

Item	Kappa index (95% confidence interval)	*p*
A	1.00 (1.00–1.00)	< 0.001
B	1.00 (1.00–1.00)	< 0.001
C	1.00 (1.00; 1.00)	< 0.001
D	#	#
E	0.89 (0.69–1.00)	< 0.001
F	0.91 (0.74 -1.00)	< 0.001
G	0.80 (0.54 -1.00)	< 0.001
H	0.93 (0.78–1.00)	< 0.001
I	1.00 (1.00–1.00)	< 0.001
J	1.00 (1.00–1.00)	< 0.001
K	1.00 (1.00–1.00)	< 0.001
L	1.00 (1.00–1.00)	< 0.001
M	0.77 (0.47–1.00)	< 0.001
N	1.00 (1.00–1.00)	< 0.001
O	1.00 (1.00–1.00)	< 0.001
P	1.00 (1.00–1.00)	< 0.001
Q	0.90 (0.72–1.00)	< 0.001
R	1.00 (1.00–1.00)	< 0.001
S	1.00 (1.00–1.00)	< 0.001
T	1.00 (1.00–1.00)	< 0.001

Note: # Both evaluators equally scored the items A to T of the 21 individuals.

The reliability analysis of the total score measures of the RULM conducted by two examinators (interrater evaluation) found an ICC of 0.998, with a 95%CI ranging from 0.995 to 0.999, indicating an extremely satisfactory reliability index (ICC > 0.95).

## DISCUSSION

The translation and cultural adaptation process of the RULM into Brazilian Portuguese is complete. All recommended steps were followed according to specific guidelines and previous studies. 16 17 19 The Brazilian version is strongly equivalent to the original. No extensive changes were necessary to the content of the manual ( **Supplementary Material S1** –available at https://www.arquivosdeneuropsiquiatria.org/wp-content/uploads/2024/12/ANP-2024.0263-Supplementary-Material-1.docx ) or the score sheet ( **Supplementary Material S2** –available at https://www.arquivosdeneuropsiquiatria.org/wp-content/uploads/2024/12/ANP-2024.0263-Supplementary-Material-2.docx ).

Only semantic and cultural adaptations were made to ensure the text was appropriate for the Portuguese language and Brazilian population. It is also important to note that no significant modifications were made to the meaning of the manual, that is to say, the initial and final positions, instructions, or scoring details. All equipment required by the original version is available in Brazil, which ensures its applicability and repeatability.

The translated RULM demonstrates robust psychometric properties, with excellent agreement and high interrater reliability. For interrater agreement, 100% concordance was obtained between the two evaluators for items A, B, C, D, I, J, K, L, N, O, P, R, S, and T.

An interesting observation was made with item D, where all responses were concentrated at score 2. In this item, the individual is asked to pick up two coins, one at a time, and hold them. All participants in our sample, even those with greater weaknesses, were able to perform the task completely, suggesting that this specific hand dexterity and manipulation item may be easier to perform compared with others, regardless of the degree of muscle weakness. Given this, it is difficult to speculate whether a larger sample might show a better distribution among scores 0, 1, and 2. Expected discrepancies between the evaluators were found only in items E, F, G, H, M, and Q. Despite this, these items continued to exhibit a good Kappa index (G and M) or an excellent Kappa index (E, F, H, and Q).

In terms of reliability, the Brazilian Portuguese version of the RULM demonstrated high reliability and adequate validity for SMA-5q individuals who can sit and/or walk, similar to the original version. 15

The RULM is an instrument revised and updated from the original ULM. One of the most significant modifications from these scales was the inclusion of weight-lifting items for stronger individuals and for reducing the risk of ceiling effect for these children. 15 In the RULM 12-month changes study, 20 a ceiling effect was observed in approximately 11% of the cohort and in ⅓ of the ambulant patients. Our study cohort did not exhibit a ceiling effect but showed higher scores than children with greater muscle weakness.

The study sample was heterogeneous, as observed in the 12-month follow-up changes study, 12 where baseline RULM scores varied between type II nonambulant and type III ambulant SMA-5q patients. Our cohort comprised individuals with SMA-5q types I, II, and III. The only type I patient, a 5-year-old nonambulatory child, scored 22. This child received treatment at 9 months of age and currently has the ability to walk with support. The greatest heterogeneity, as expected, was observed among type-II individuals, who ranged from 4 to 15-years-old and scored between 15 and 37 points. The only ambulatory type II patient, a 4-year-old child, achieved a score of 32. The patient with the lowest score in this group was 9-years-old. Among type-III SMA-5q individuals, we observed the widest age variation, ranging from 9 to 56 years. The majority (6/8) scored above 30 points, with 3 of them being ambulatory.

As a limitation, we acknowledge this study's relatively small sample size, despite recognizing the difficulty in recruiting patients with neuromuscular diseases.

In conclusion, the Brazilian Portuguese version of the RULM enables its use for patients with SMA-5q, both in clinical practice and trials conducted in the country.
